# [^153^Sm]Samarium-labeled FAPI-46 radioligand therapy in a patient with lung metastases of a sarcoma

**DOI:** 10.1007/s00259-021-05273-8

**Published:** 2021-03-17

**Authors:** Clemens Kratochwil, Frederik L. Giesel, Hendrik Rathke, Rebecca Fink, Katharina Dendl, Jürgen Debus, Walter Mier, Dirk Jäger, Thomas Lindner, Uwe Haberkorn

**Affiliations:** 1 Department of Nuclear Medicine, Heidelberg University Hospital, INF 400, 69120 Heidelberg, Germany; 2 Department of Radiation Oncology, Heidelberg University Hospital, Heidelberg, Germany; 3 Department of Medical Oncology, Heidelberg University Hospital and National Center for Tumor Diseases (NCT), Heidelberg, Germany; 4 Cooperation Unit Nuclear Medicine, German Cancer Research Center (DKFZ), Heidelberg, Germany; 5 Translational Lung Research Center Heidelberg (TLRC), German Center for Lung Research (DZL), Heidelberg, Germany

## Image of the month

Fibroblast activation protein is overexpressed by cancer-associated fibroblasts (CAFs) in the stroma of several tumor entities and provides anti-immunogenic effects [1]. It can be targeted with radiolabeled small-molecule inhibitors (FAPIs) [2].

This image demonstrates a patient with progression of lung metastatic, fibrous spindle cell soft tissue sarcoma. Primary tumor located between bladder and rectum as well as early generations of oligo-focal metastases had previously been treated by resection and external-beam radiotherapy. In systemic stage, mutanom-based vaccination [3], cyclophosphamide, and pazopanib had already been used but the patient was considered inappropriate for standard chemotherapy with anthracyclines. An interdisciplinary tumor conference considered experimental FAPI-RLT a promising option for this therapy-refractory patient to serve as a “can opener” for succeeding immunotherapy.

FAPI-PET/CT demonstrated target positive tumor phenotype (a). Due to the relatively short biological tumor half-life of quinoline-based FAPI-46 [1], it was labeled with short physical half-life (46.3 h) ^153^Sm. Emission scans during therapy demonstrate tumor targeting up to 44 h p.i. and rapid clearance from normal organs (b). Three cycles with cumulative 20 GBq ^153^Sm- and 8GBq Y-90-FAPI-46 (^153^Sm was not available with sufficiently high specific activity) were well tolerated and achieved stable disease for 8 months (c). Next treatment lines were pembrolizumab, experimentally enhanced with oncolytic parvovirus [4], and nab-paclitaxel. Under both therapies, the patient progressed after only 3 months.

One explanation for the clinical activity of ^90^Y/^153^Sm-FAPI-46 in this particular case might be FAP expression in both CAFs and sarcoma tumor cells [5], which is unfortunately not the case in other tumor entities. Of course, one case is no proof of general efficacy but obviously this image encourages further studies of FAPI-RLT against soft tissue sarcoma. However, due to technical issues related to ^153^Sm, e.g., regarding specific activity and contamination with ^154^Eu, additional short physical half-life isotopes should be evaluated as alternative options.

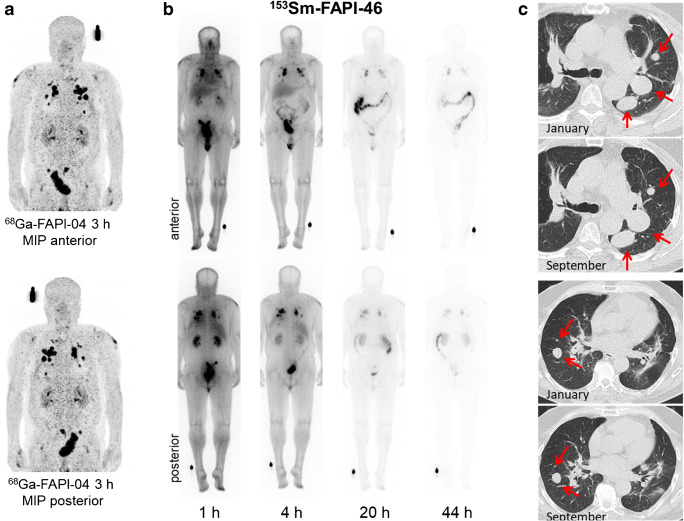



## Data Availability

Not applicable
